# Differential Expression Profiles and Potential Intergenerational Functions of tRNA-Derived Small RNAs in Mice After Cadmium Exposure

**DOI:** 10.3389/fcell.2021.791784

**Published:** 2022-01-03

**Authors:** Ling Zeng, Jinzhao Zhou, Yanwei Zhang, Xiaofei Wang, Mei Wang, Ping Su

**Affiliations:** ^1^ Institute of Reproductive Health, Tongji Medical College, Huazhong University of Science and Technology, Wuhan, China; ^2^ Reproductive Medicine Center, Zhongnan Hospital of Wuhan University, Wuhan, China

**Keywords:** cadmium, sperm tsRNA, intergenerational epigenetic inheritance, cell membrane system, mitochondrion and lysosome

## Abstract

Cadmium (Cd) is a toxic heavy metal and ubiquitous environmental endocrine disruptor. Previous studies on Cd-induced damage to male fertility mainly focus on the structure and function of testis, including cytoskeleton, blood-testis barrier, and steroidogenesis. Nevertheless, to date, no studies have investigated the effects of Cd exposure on sperm epigenetic inheritance and intergenerational inheritance. In our study, we systematically revealed the changes in sperm tRNA-derived small RNAs (tsRNA) profiles and found that 14 tsRNAs (9 up-regulated and 5 down-regulated) were significantly altered after Cd exposure. Bioinformatics of tsRNA-mRNA-pathway interactions revealed that the altered biological functions mainly were related to ion transmembrane transport, lipid metabolism and cell membrane system. In addition, we focused on two stages of early embryo development and selected two organs to study the impact of these changes on cell membrane system, especially mitochondrion and lysosome, two typical membrane-enclosed organelles. Surprisingly, we found that the content of mitochondrion was significantly decreased in 2-cell stage, whereas remarkably increased in the morula stage. The contents of mitochondrion and lysosome were increased in the testes of 6-day-old offspring and livers of adult offspring, whereas remarkably decreased in the testes of adult offspring. This provides a possible basis to further explore the effects of paternal Cd exposure on offspring health.

## Introduction

In recent years, the enigma of how environmental exposure induces phenotypic changes across multiple generations of offspring has aroused much interest. Environmental inputs, including an unhealthy diet (Chen et al., 2016; Sharma et al., 2016), toxin exposure (Anway et al., 2005; Salian et al., 2009; Wolstenholme et al., 2012), obesity (Ng et al., 2010; Saikia et al., 2014), environmental enrichment (a combination of physical and mental exercise) (Benito et al., 2018), mental stress (de Castro Barbosa et al., 2016) and psychological trauma (Yehuda et al., 2005; Gapp et al., 2014), can reshape the sperm epigenome, especially the small non-coding RNA (sncRNA) expression profile, and affect offspring phenotypes (Zhang et al., 2019).

Cadmium (Cd) is a toxic heavy metal and ubiquitous environmental endocrine disruptor (EDC), which is widely present in contaminated foods, water, soil and air at low concentrations (Sirot et al., 2008; Järup and Åkesson, 2009; Satarug, 2018; Genchi et al., 2020; Shi et al., 2020). Notably, smoking is an increasingly important route to Cd exposure (Faroon et al., 2012; Taha et al., 2018). Continuous low-dose Cd exposure can lead to its slow accumulation in human body, with a long half-life of 25–30 years. Studies have shown that environmental Cd can affect male fertility through various pathways, seriously threatening male reproductive health (Mathur and D’cruz, 2011; Ji et al., 2012; Cupertino et al., 20172017a; Wu et al., 2017; Wang et al., 2017). Previous studies on Cd-induced damage to male fertility mainly focus on the testis physiology, especially in cytoskeleton (Egbowon et al., 2016; Venditti et al., 2021), blood-testis barrier (Chen et al., 2018; Fang et al., 2020; Venditti et al., 2021) and steroidogenesis (Cupertino et al., 20172017b; Habib et al., 2019; Shi and Fu, 2019). As a result, spermatogenesis disruption leads to the abnormalities of sperm concentration and motility. Nevertheless, to date, there are no studies focusing on sperm epigenetic inheritance after Cd exposure. Whether Cd could affect sperm tsRNAs expression profile and further affect offspring health remains to be elucidated.

Previous studies suggest that epigenetic markers in germ cells, which include DNA methylation, histone modifications, and sncRNAs, are capable of both responding to parental environmental factors and affecting offspring phenotypes. These epigenetic markers are potentially positioned to mediate intergenerational or transgenerational epigenetic inheritancec (Skinner, 2008; Skvortsova et al., 2018; Perez and Lehner, 2019). Recently, the research of tsRNAs has undergone a renaissance, demonstrating that sperm tsRNAs could mediate intergenerational inheritance of acquired traits as a type of paternal epigenetic information carrier (Yan and Zhai, 2016). According to published RNA-sequencing data, the sncRNA population in mature mouse sperm is mainly composed of tsRNAs, while microRNAs (miRNAs) and Piwi-interacting RNAs (piRNAs) accounted for a small proportion (Peng et al., 2012; Chen et al., 2016; Sharma et al., 2016; Zhang et al., 2019). Interestingly, sperm tsRNAs are not directly derived from the testes, but from extracellular vesicles (EVs) produced by epididymal epithelial cells during post-testicular sperm maturation (Sharma et al., 2016).

Recent studies show that sperm tsRNA expression profiles are altered in mice fed a low-protein diet (LPD) (Sharma et al., 2016), or a high-fat diet (HFD) (Chen et al., 2016), in rats fed an HFD (de Castro Barbosa et al., 2016), and in obese humans (Donkin et al., 2016), suggesting that sperm tsRNAs can function as sensitive markers of environmental stimuli. Moreover, offspring from zygotes injected with sperm RNAs, total RNAs or even only tsRNAs, from mice fed a HFD showed impaired glucose tolerance and insulin secretion (Chen et al., 2016). The deletion of DNA methyltransferase 2 (Dnmt2), a highly conserved DNA methyltransferase, affected the expression profile of sncRNAs in sperm and prevented the increase of RNA m5C and m2G modifications in 30-40nt sperm RNA fragments induced by a HFD. Specifically, injecting total RNAs from the sperm of mice fed a HFD into zygotes resulted in abnormal glucose tolerance, while injecting RNAs extracted from the sperm of Dnmt2^−/−^ HFD-fed animals did not induce similar phenotypes (Zhang et al., 2018). These findings provide evidence that sperm tsRNAs, along with tsRNAs modifications, are involved in the intergenerational inheritance.

Therefore, we aim to investigate sperm tsRNA expression profiles and preliminarily determine the potential functional roles of candidate tsRNAs in the offspring after paternal Cd exposure. In the present study, we studied the sperm tsRNA expression profiles of Cd-exposured mice by RNA-sequencing technologies and validated 14 tsRNAs with quantitative real-time PCR (qRT-PCR). After that, the potential effects on their offspring were analyzed. These findings may provide new clues for studying the effects of Cd exposure on the health of offspring.

## Materials and Methods

### Animals and Experimental Groups

All the animal procedures were approved by the Institutional Animal Care and Use Committee of Tongji Medical College, Huazhong University of Science and Technology. All experiments with mice were conducted ethically according to the Guide for the Care and Use of Laboratory Animal guidelines (**S2547**). 8 weeks adult male C57BL/6J mice were housed for 1 week before the study in a temperature (22–25°C) and humidity controlled (50% relative humidity) animal facility with 12 h light/dark cycles. The animals had free access to food and water. 30 mice were randomly assigned into two groups. Refer to our previous study (Wang et al., 2020a; Wang et al., 2020b), the control group was treated with 0.9% NaCl (equivalent to receiving 0 mg/kg of body weight of CdCl_2_), and the treatment group was intraperitoneally injected with CdCl_2_ at the concentration of 1.0 mg/kg of body weight for 5 weeks every other day. CdCl_2_ was purchased from Sigma Chemical Co. (St. Louis, MO, United States).

### Cadmium Content in the Testis

As previously reported (Wang et al., 2020b; Zeng et al., 2021), Cd content in testis was detected by graphite furnace atomic absorption spectrometry (GFAAS). The samples were baked and weighed. 3 ml nitric acid and 0.5 ml perchloric acid were added to dissolve the samples into a flowing liquid. And then the samples were baked on a 280°C hotplate for 1 h. After the samples cooled, 5 ml pure water was added to the volume. The operating conditions for GFAAS require atomization at 1,600°C for Cd after a heating phase (110°C and then 1,308°C) and pyrolysis at 500°C for Cd. The wavelength was 228.80 nm for Cd.

### Histopathological Analyses of the Testis

Unilateral testis of 5 mice in each group were used for histopathological analyses of the testis. The testes were fixed in Bouin’s solution following dissection and then paraffin-embedded. The paraffin blocks were then sectioned by a Lycra paraffin slicer into 4-μm-thick sections, and at least 2 sections for each paraffin blocks. The testicular sections were immediately de-waxed, rehydrated, and stained with hematoxylin and eosin (HE). The images were captured using an inverted microscope.

### Sperm Parameter Analysis

Basic semen analysis followed the recommendations of the World Health Organization (WHO) 2010 manual for the examination of human semen. The sperm concentration was assessed using an improved Neubauer hemocytometer. Motility measurements were performed to record the progressive sperm count per 200 sperm under a microscope.

### Mature Sperm Collection

Animals were sacrificed by cervical dislocation. As previously reported (Chen et al., 2016), mature sperm were isolated from cauda epididymis of male mice and processed for RNA extraction. In brief, sperm were released from cauda epididymis into 1ml Nutrient Mixture F-10 Ham (Sigma-Aldrich, N6013-500ML) maintained at 37*°*C for 30 min incubation. After that, sperm were filtered with 100um and then 40°µm cell strainer to remove the tissue debris. The sperm were then treated with somatic cell lysis buffer (0.1% SDS, 0.5% Triton X-100 in DEPC H_2_O) for 30 min on ice to eliminate somatic cell contamination. The sperm were pelleted by centrifugation at 600 g for 5 min. After removal of suspension, the sperm pellet was resuspended and washed twice in 10ml of phosphate‐buffered saline (PBS) then pelleted at 600 g for 5 min.

### RNA Extraction

The total RNA was isolated using TRIzol LS Reagent (Invitrogen Corp., Carlsbad, CA) based on the manufacturer’s instructions. Before the sequencing experiment, agarose gel electrophoresis was used to check the integrality of total RNA samples, and then the samples were quantified on the NanoDrop ND-1000 instrument.

### Small RNA Library Preparation and Sequencing

In order to remove RNA modifications that interfered with small RNA-seq library construction, we used the commercial kit (NEBNext^®^ Multiplex Small RNA Library Prep Set for Illumina^®^) for tRF & tiRNA-seq library preparation, including 3′-aminoacyl (charged) deacylation to 3′-OH for 3adaptor ligation, 3′-cP (2′,3′-cyclic phosphate) removal to 3′-OH for 3′adaptor ligation, 5′-OH (hydroxyl group) phosphorylation to 5′-P for 5′-adaptor ligation, m1A and m3C demethylation for efficient reverse transcription. Pretreated total RNA of each sample was taken for tRF & tiRNA-seq library preparation. The prepared tRF & tiRNA-seq libraries were finally absolutely quantified using Agilent 2,100 BioAnalyzer (Agilent Inc., MD, United States), then sequenced using Illumina NextSeq 500 (Illumina, CA, United States). For standard small RNA sequencing, the sequencing type was 50-bp single-read.

### Sequencing Data Analysis

The raw sequencing data that passed the Illumina chastity filter were used for the following analysis. Sequencing quality are examined by FastQC software (v0.11.7) and 5′, 3′-adaptor bases were trimmed by cutadapt software (1.17). The trimmed reads are aligned allowing for 1 mismatch only to the mature tRNA sequences, and then reads that do not map were aligned to precursor tRNA sequences with bowtie software (v1.2.2). The remaining reads were aligned allowing for 1 mismatch only to miRNA reference sequences with miRDeep2 (2.0.0.8) (Friedländer et al., 2012). The expression profiling of tRF & tiRNA and miRNA can be calculated based on counts of reads mapped and normalized as counts per million (CPMs) of the total aligned tRNA reads. The differentially expressed tRFs & tiRNAs and miRNAs were screened based on the count value with R package DEseq2 (Love et al., 2014). When comparing the two groups for profile differences (Cd group vs. Ctrl group), the ‘‘fold change (FC),’’ i.e., the ratio of the group averages between the groups was computed for each tsRNA. An |log2 (FC)| *>* 1.5 and a *p*-value *<* 0.05 were considered as a significantly different expression, and the tsRNAs with such values were chosen for the next analysis. Pie plots, Venn plots, hierarchical clustering, scatter plots, and volcano plots were performed in an R environment for statistical computing and graphical presentation of the expressed tsRNAs and miRNAs.

### Bioinformatic Prediction

The 14 significantly differentially expressed tsRNAs (|log2 (FC)| *>* 1.5 and *p*-value *<* 0.05) selected from the sequencing data were analyzed using bioinformatic methods. The candidate selection was based on several points: 1) higher FC and lower *p*-value; 2) higher CPM in both groups and balanced expression of each mouse in each group; 3) Referring to previous publications to achieve more evidence of similar subtype of tsRNA. First, in terms of target prediction of the candidate tsRNAs, the tsRNAs contained some seed sequences that could match the crosslink-centered regions of the target mRNAs (Kim et al., 2017). Mounting evidence has strongly suggested that tsRNAs could act as miRNAs, silencing the target mRNA *via* complementary base pairing (Kumar et al., 2014). According to a previous study (Li et al., 2019), two common algorithms were used to predict the target genes, including TargetScan (Lewis et al., 2005; Grimson et al., 2007; Friedman et al., 2009; Jan et al., 2011), and miRanda (Enright et al., 2003). Notably, to reduce the false-positive results, only the genes predicted by all two algorithms were considered as target genes of the tsRNAs.

Second, to assign the biological annotation of the target genes, enrichment analyses were performed. We used the package clusterProfiler (3.18.1) (Yu et al., 2012) for Gene Ontology (GO) and Kyoto Encyclopedia of Genes and Genomes database (KEGG) pathway annotations. GO enrichment analysis was used to reveal the biological process, cellular component, and molecular function of the target genes. KEGG enrichment analysis was used to identify the significant pathways. A *p <* 0.05 indicated the significance of the GO and KEGG enrichment pathways. The false discovery rate was calculated to correct the *p*-values.

As for differentially expressed miRNAs, we used R package multiMiR (0.98.0.2) (Ru et al., 2014) to predict the target genes. Furthermore, we used the package clusterProfiler (3.18.1) (Yu et al., 2012) for GO and KEGG pathway annotations.

### qRT-PCR Assay

TsRNA acts in a manner similar to miRNA, recognizing binding sites on target mRNA 3 ′-UTR through its seed sequence (Li et al., 2019). The differential genes were classified according to the seed sequence, and 1-2 tsRNAs were selected from the differential genes with the same seed sequence for primer design. Afterwards, cDNA synthesis and qPCR detection were done using All-in-OneTM miRNA qRT-PCR Detection Kit (No.QP015, GeneCopoeia, Inc., MD, United States) according to manufacturer’s instructions. The temperature program consisted of an initial single cycle of 10 min at 95°C, followed by 40 cycles of 10 s at 95°C, 20 s at 58°C and 30 s at 72°C. PCR reactions were implemented using a StepOnePlus™ realtime PCR system (Version 2.2.2, Applied Biosystems, MA, United States). The cycle threshold (Ct) values were recorded, and relative expression of the target genes was calculated using the 2^–ΔΔCT^ method. RNU6 was used as the internal control to normalize the data. PCR primers were synthesized by Sangon Biotech (China). The primer sequences are listed in Supplementary Table S1. All experiments were repeated three times.

### Collection of Embryos and Immunofluorescence

We used 5 male mice in each group at a time to co-house with unexposed female mice in the afternoon. The housed ratio of male to female was 1:2. The morning of the day on which a vaginal plug was detected was designated embryonic day 0.5 (E0.5). Embryos at different stages of development (E1.5 through E3.5) were collected by flushing oviducts or the uterus with HEPES-buffered medium 2 (M2; Sigma). Embryos were fixed immediately for 30 min with PBS containing 3% paraformaldehyde and washed in 0.1% BSA/PBS. After blocking with 0.1% Triton X-100/1% BSA in PBS, embryos were incubated with anti-VDAC1/2 antibody (10866-1-AP, 1:100, Proteintech, China) at 4°C overnight. After washing with 0.1% BSA/PBS 3 times for 10 min each, embryos were incubated with the secondary antibody (SA00013-2, 1:200, Proteintech, China). The nucleiwere counter-stained with DAPI in the dark. The images were acquired using a confocal microscope (ZEISS LSM 780) in Tongji Medical College, Huazhong University of Science and Technology. The same microscope settings were maintained between groups and replicates. During imaging, z-stack was used to stratify the embryos and Orthogonal Projection was used to get the merged image. Afterwards, those merged images were used for statistical analysis through ZEN 3.2 (blue edition).

### Western Blot Analysis

Total proteins from 6 day-old progeny testes, 16 week-old progeny livers and testes were extracted immediately after the mice were sacrificed. The protein concentration in the extracts was determined using a BCA protein assay kit (BL521A, Biosharp, China). Proteins were denatured, separated via sodium dodecyl sulfate-polyacrylamide gel electrophoresis (SDS-PAGE), and transferred to a polyvinylidene fluoride (PVDF) membrane, which was then blocked in 5% non-fat milk at room temperature for 2 h. The membranes were then washed three times using Tris buffered saline tween (TBST) for 10 min each. Subsequently, the membranes were incubated with primary antibodies according to the manufacturer’s instructions, including VDAC1/2 (10866-1-AP, 1:1,000, Proteintech, China), LAMP2 (27823-1-AP, 1:1,000, Proteintech, China), and β-Actin (66009-1-Ig, 1:1,000, Proteintech, China). Next, the membranes were rinsed and incubated with horseradish peroxidase (HRP)-conjugated secondary antibodies (HRP-conjugated Affinipure Goat Anti-Rabbit IgG(H + L), SA00001-2, 1:4,000; HRP-conjugated Affinipure Goat Anti-Mouse IgG(H + L), SA00001-1, 1:4,000; both from Proteintech, China). The specific immunoreactive protein bands were developed using ECL reagent (G2014-50ML, Servicebio, China).

### Immunofluorescence Microscopy

The progeny livers and testes were paraffin-embedded and cut into 4-µm sections using a microtome (Leica, Germany). Each section was de-waxed, followed by antigen retrieval by boiling in 10 mM sodium-citrate buffer pH 6.0 for 10 min. After blocking with 5% goat serum (GS), the primary antibody (VDAC1/2, 10866-1-AP, 1:100; LAMP2, 27823-1-AP, 1:100; both from Proteintech, China) was added to the sections and incubated at 4°C overnight, followed by washing with 5% GS 3 times for 10 min each, and incubation with the secondary antibody (SA00013-2, 1:200 or SA00013-3, 1:200, both from Proteintech, China). The nuclei were counter-stained with DAPI in the dark. The images were acquired using a confocal microscope (ZEISS LSM 780). The fluorescence intensity was analyzed using ZEN 3.2 (blue edition).

### Transmission Electron Microscopy

Progeny liver and testis samples were fixed with 2.5% glutaraldehyde for 2 h at 4°C, post-fixed with 1% osmium tetroxide, and embedded in Epon 812. Blocks were cut into semi-thin sections and stained with methanolic uranyl acetate and lead citrate. The liver and testicular ultrastructure was investigated by transmission electron microscopy (TEM) (JEM 1200-EX; Hitachi, Ltd, Tokyo, Japan) at 80 kV.

### Statistical Analyses

GraphPad Prism 8 (GraphPad Inc., CA, United States) was used for the visualization of graphs and data analysis. All data are presented as the means ± standard deviations (SD). Differences between groups were analyzed using Student’s t-test or single factor analysis of variance (one-way ANOVA). Differences with *p* values of less than 0.05 were considered statistically significant.

## Results

### Effects of Cd Exposure on Male Reproductive System in Mice

**FIGURE 1:**
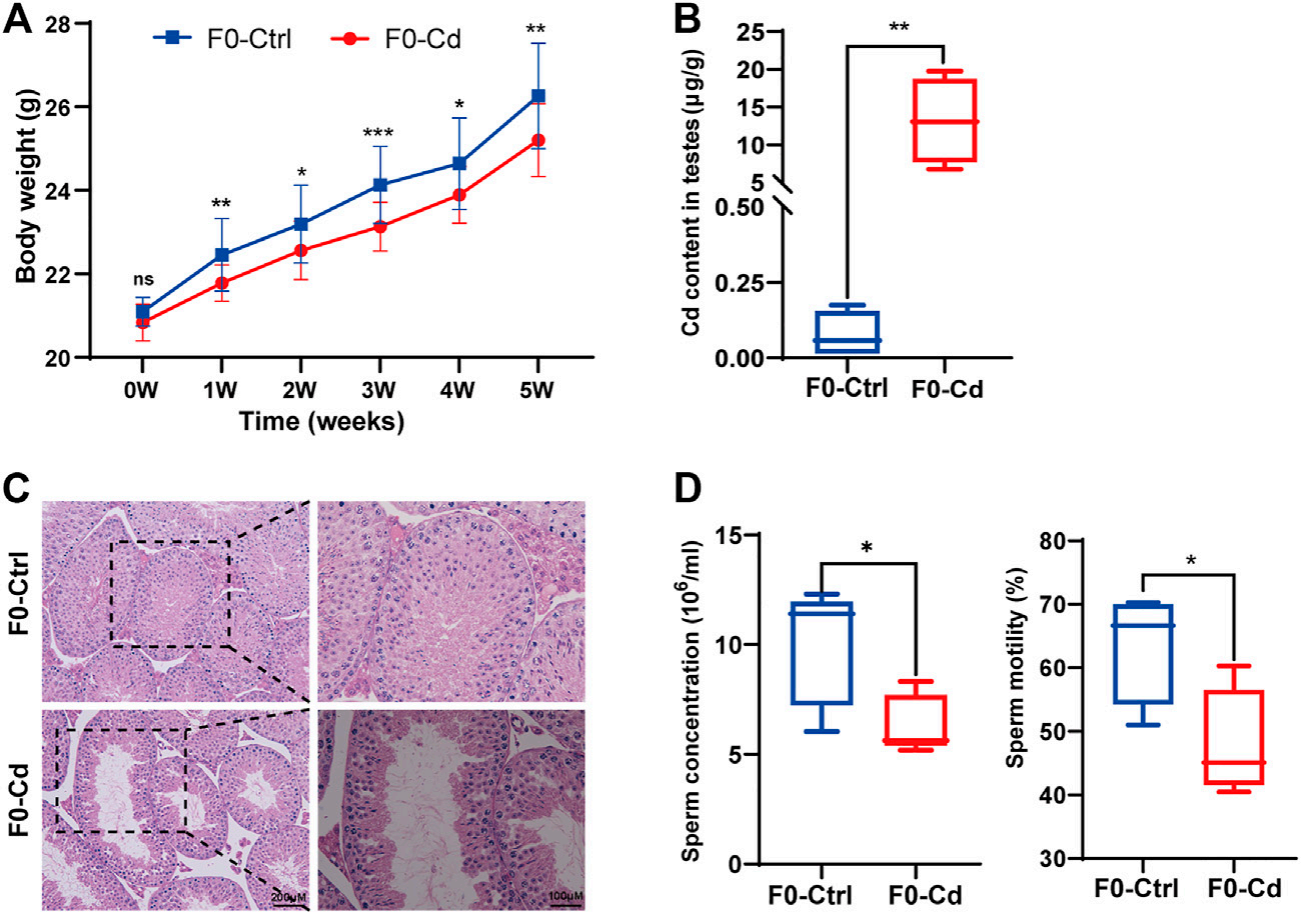
Effects of Cd exposure on male reproductive system in mice. **(A)** Body weight of F0 males (*n* = 15). **(B)** Cd content in the testes of F0 mice (*n* = 5). **(C)** HE results of the testis in F0 mice. **(D)** Sperm concentration and sperm motility in F0 mice (*n* = 5). All results are expressed as the mean ± SD. Labeled asterisk represents statistical significance compared with the respective control group. *p < 0.05, **p < 0.01 and ***p < 0.001. ns, no significant difference.

Adult male C57BL/6 J mice were treated with Cd at a dose of 1 mg/kg for 5 weeks every other day. Body weights were gained more slowly in the Cd-treated group (Figure 1A). After Cd exposure, the Cd content in testis was remarkably increased (*p* < 0.01; Figure 1B). Cd resulted in testicular morphometric injury. As shown in Figure 1C, the structure of the seminiferous tubules in F0-Cd was disrupted and the tubes were loosely arranged, even exhibiting severe vacuolization. Significantly, Cd reduced sperm motility and counts in the epididymis, compared with the control group (Figure 1D). These data indicated that Cd induced male reproductive dysfunction in mice.

### Effects of Cd Exposure on Sperm tsRNAs Expression Profile

In eukaryotic cells, tsRNAs are produced through specific cleavage of precursor tRNAs (Pre-tRNAs) and mature tRNAs by different endonucleases. The tsRNAs generated from mature tRNAs can be divided into two main types: tRNA halves (or tRNA derived stress-induced RNAs, also known as tiRNAs), which are the product of angiogenin (ANG) cleavage of tRNA at the anti-codon site during stress (Elkordy et al., 2018), and smaller tRNA fragments (tRFs), which are miRNA-like fragments that interact with Argonaute protein (Cole et al., 2009; Lee et al., 2009; Martinez et al., 2017; Lyons et al., 2018). The tRNA halves, which are 31–40 nucleotides (nt) long, have two subtypes: 5′-tRNA halves (tiRNA-5) and 3′-tRNA halves (tiRNA-3). The tRFs are 14–30 nt long and mainly consist of three subtypes: tRF-5, tRF-3, and tRF-1. Additionally, tsRNAs derived from pre-tRNAs can be divided into two types according to the presence of sequences derived from the 5′ leader or 3’ trailer (Anderson and Ivanov, 2014).

**FIGURE 2:**
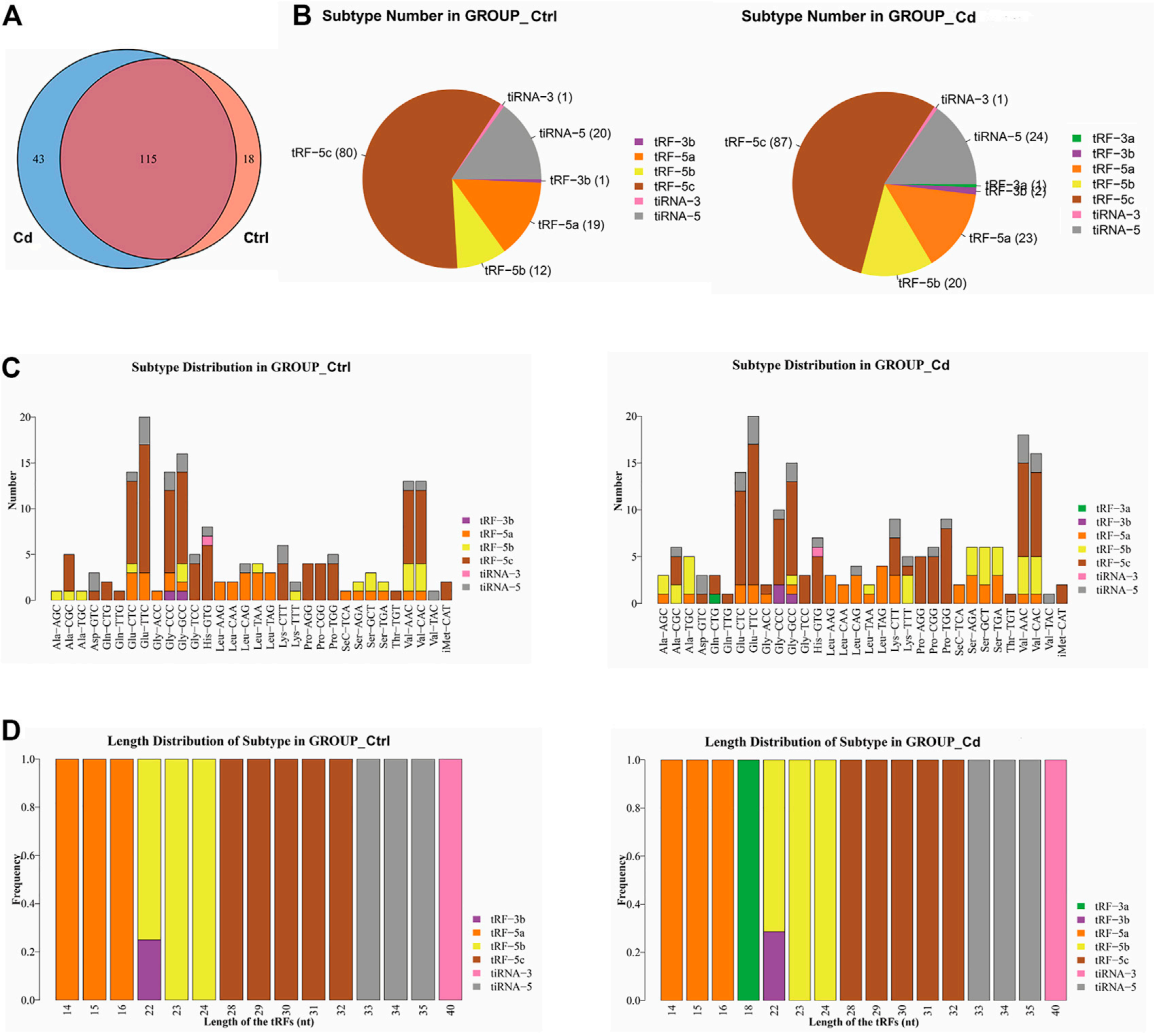
Effects of Cd exposure on sperm tsRNAs expression profile. **(A)** Venn diagram of the number of tsRNAs. The pie plot shows the number of tsRNAs which were expressed in both of the groups and also the number of specific expressed tsRNAs in Ctrl or Cd group. **(B)** Pie charts for all tsRNAs of each group using all uniquely expressed tsRNAs. The pie chart show the distribution of the number for each subtype tsRNAs which the average CPM of the group is not less than 20. **(C)** The stacked bar charts stack bars that represent different tRNA isodecoders on top of each other, respectively. The height of the resulting bar shows the combined result of tRNA isodecoders. **(D)** The stacked bar charts represent the length distribution of tsRNA subtype for each group. CPM: counts per million of total aligned reads.

In order to explore the effect of Cd exposure on sperm tsRNA expression profile, we used Illumina NextSeq 500 for tsRNAs sequencing. After the quality filtering, we identified 115 commonly expressed tsRNAs, 18 tsRNAs specifically expressed in the control group, and 43 tsRNAs specifically expressed in the Cd group (Figure 2A). Next, as shown in Figure 2B, a pie chart was performed of each tsRNA subtype, indicating that most sperm tsRNAs were generated from mature tRNAs. In those tsRNAs, the number of each tsRNA subtype were quite different. Overall, the number of tRF-5 (including tRF-5a, tRF-5b, tRF-5c) was increased in Cd group, as compared to the control group. Interestingly, tRF-3a specially appeared after Cd treatment.

As we know, tRNA isodecoders share the same anticodon but have differences in their body sequence (Geslain and Pan, 2010). The number of tsRNA subtypes can be counted against tRNA isodecoders. In the stacked bar charts (Figure 2C), stack bars respectively represent different tRNA isodecoders on top of each other. The height of the respective bar shows the gross result of tRNA isodecoders. Next, as shown in Figure 2D, stack bars respectively represent the frequency of subtypes against the length of the tsRNA. Above all, the most significant difference in the isodecoders or frequency of subtypes between the two group was that 18-nt tRF-3a (Gln-CTG) specially appeared after Cd treatment.

**FIGURE 3:**
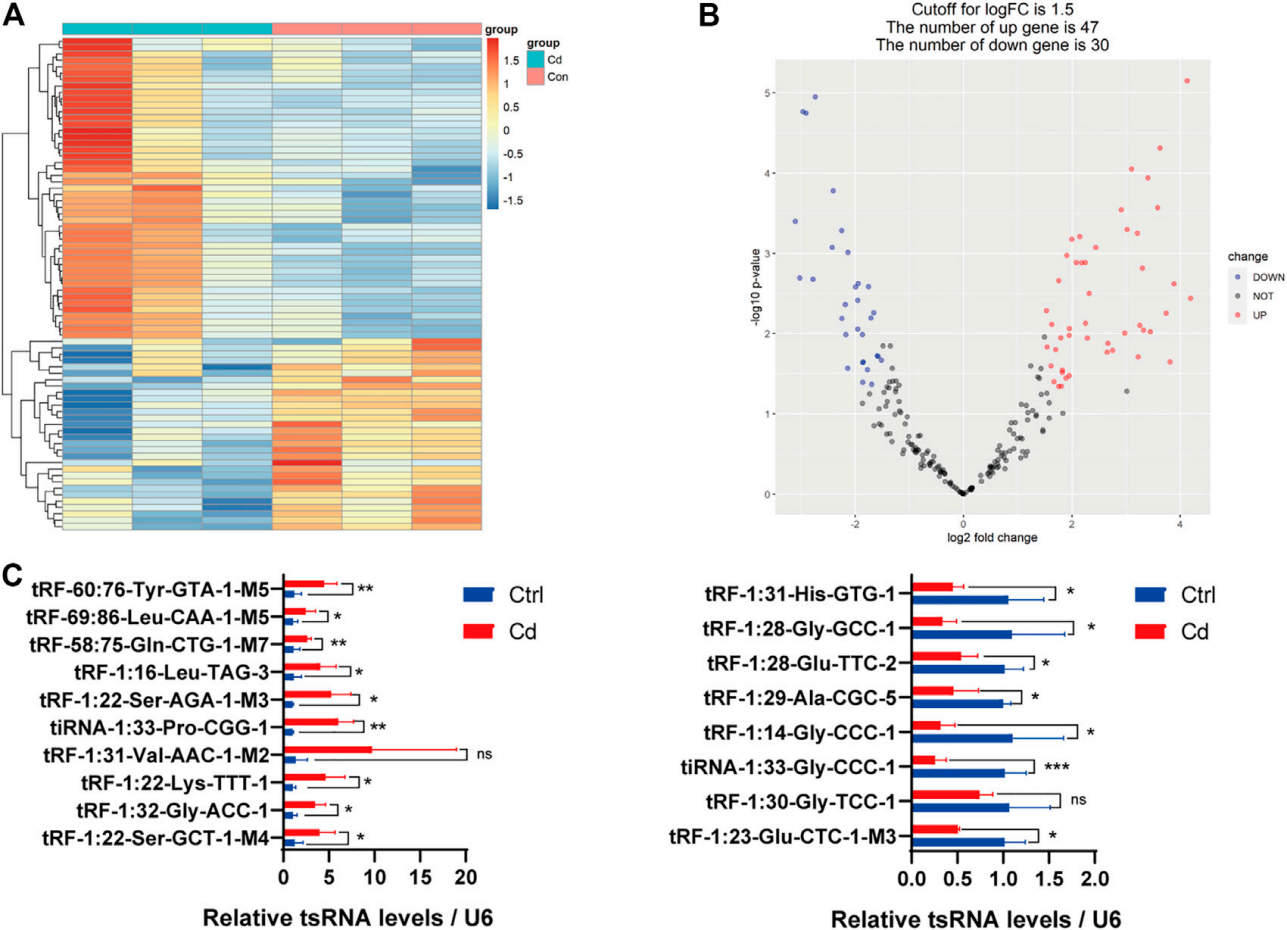
Differential expression analysis of tsRNAs between the two groups. **(A)** The heat-map for the differentially expressed tsRNAs between the two groups. The color in the panel represents the relative expression level (log2-transformed). The colored bar at the top panel shows the different groups. **(B)** The volcano plot is constructed by plotting -log10 (*p*-value) on the *y*-axis, and tsRNAs expression log2 (fold change) between the two experimental groups on the *x*-axis, providing a visualization method to perform a quick visual identification of the differentially expressed tsRNAs. **(C)** The validation of the 10 up-regulated tsRNAs and 8 down-regulated tsRNAs using qRT-PCR, respectively (*n* = 6). All results are expressed as the mean ± SD. Labeled asterisk represents statistical significance compared with the respective control group. *p < 0.05, **p < 0.01 and ***p < 0.001. ns, no significant difference.

In addition, we identified that a total of 53 tsRNAs were significantly differentially expressed between the two groups: 32 were significantly up-regulated, whereas 21 were significantly down-regulated (Cd vs. Control; |Log2FC| *>* 1.5; *p <* 0.05). The experimental data on the top 10 upregulated and downregulated tsRNAs ranked by fold change are presented in Table 1. As shown in Figure 3A, heat map was performed using the significantly differentially expressed tsRNAs, indicating a distinguishable tsRNA expression profiling among the samples. Next, volcano plot was constructed using the fold change values and *p*-values to visualize the differential expression between the two different conditions (Figure 3B).

**TABLE 1:** The top 10 up-regulated and down-regulated tsRNAs ranked by logFC after Cd exposure.

tsRNA_ID	Type	tRFdb_ID	Length	logFC	padj	Regulation
tRF-59:75-Pro-AGG-1-M4	tRF-3a	-	17	4.186784	0.024808	Up
tiRNA-1:34-Glu-TTC-3	tiRNA-5	-	34	4.124974	0.001015	Up
tRF-69:86-Leu-CAA-1-M5	tRF-3a	3018a	18	3.881438	0.01871	Up
tRF-1:15-Ala-AGC-1-M3	tRF-5a	-	15	3.739387	0.033242	Up
tRF-1:22-Ser-AGA-1-M3	tRF-5b	-	22	3.625968	0.002213	Up
tiRNA-1:34-Glu-CTC-2	tiRNA-5	-	34	3.58281	0.006484	Up
tRF-58:75-Pro-AGG-1-M4	tRF-3a	3003a	18	3.443005	0.045347	Up
tiRNA-1:34-Glu-CTC-1	tiRNA-5	-	34	3.400312	0.003704	Up
tRF-58:75-Gln-CTG-1-M7	tRF-3a	3007a	18	3.320385	0.044557	Up
tiRNA-1:34-Ala-CGC-5	tiRNA-5	-	34	3.300042	0.0144	Up
tRF-1:28-His-GTG-2	tRF-5c	-	28	−2.24893	0.036493	Down
tiRNA-1:34-His-GTG-2	tiRNA-5	-	34	−2.25266	0.00901	Down
tRF-1:30-Gly-TCC-1	tRF-5c	-	30	−2.41074	0.004686	Down
tRF-1:31-Gly-TCC-1	tRF-5c	-	31	−2.42949	0.010598	Down
tRF-1:30-His-GTG-2	tRF-5c	-	30	−2.73888	0.001015	Down
tRF-1:22-Gly-GCC-2-M3	tRF-5b	5004a	22	−2.78403	0.018232	Down
tRF-1:29-His-GTG-2	tRF-5c	-	29	−2.912	0.001015	Down
tRF-1:24-Gly-GCC-2-M3	tRF-5b	-	24	−2.96808	0.001015	Down
tRF-1:16-Gly-CCC-1	tRF-5a	-	16	−3.02596	0.018232	Down
tRF-1:23-Glu-CTC-1-M3	tRF-5b	5022b	23	−3.10811	0.008184	Down

Subsequently, we detected the expression level of the 18 candidate tsRNAs using qRT-PCR to comfirm the tsRNA-Seq results (Figure 3C; Supplementary Table S1). As a result, compared with the control group, 9 candidate tsRNAs (tRF-60:76-Tyr-GTA-1-M5, tRF-69:86-Leu-CAA-1-M5, tRF-58:75-Gln-CTG-1-M7, tRF-1:16-Leu-TAG-3, tRF-1:22-Ser-AGA-1-M3, tiRNA-1:33-Pro-CGG-1, tRF-1:22-Lys-TTT-1, tRF-1:32-Gly-ACC-1 and tRF-1:22-Ser-GCT-1-M4) were significantly up-regulated in the Cd group, while 7 candidate tsRNAs (tRF-1:31-His-GTG-1, tRF-1:28-Gly-GCC-1, tRF-1:28-Glu-TTC-2, tRF-1:29-Ala-CGC-5, tRF-1:14-Gly-CCC-1, tiRNA-1:33-Gly-CCC-1 and tRF-1:23-Glu-CTC-1-M3) were significantly down-regulated in the Cd group.

In general, based on the sequencing data and qRT-PCR validation, it seems reasonable to conclude that Cd exposure may affect sperm tsRNA expression.

### Bioinformatics Analysis

**FIGURE 4:**
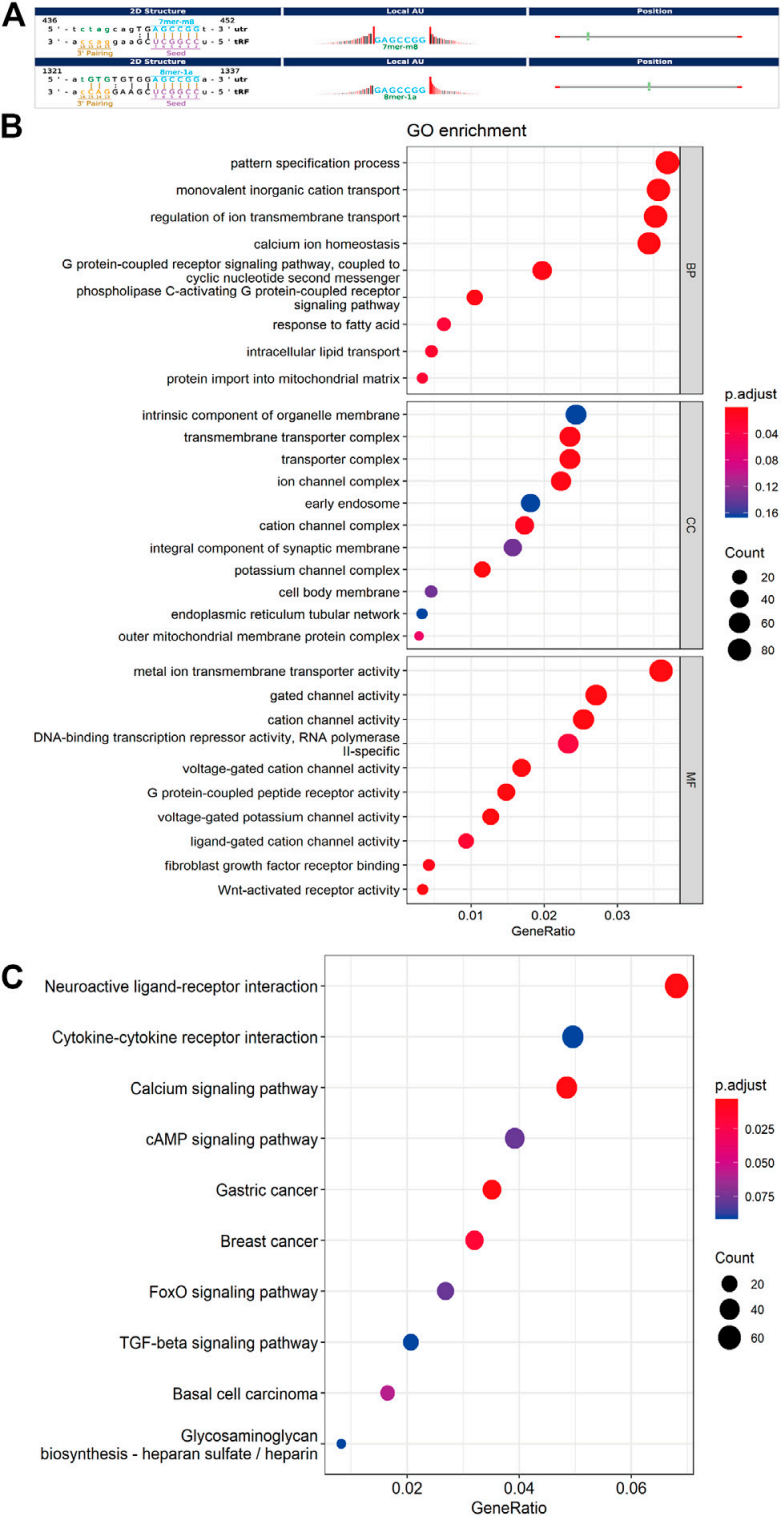
Bioinformatic prediction and functional enrichment analysis of differentially expressed tsRNAs between the two groups. **(A)** The 2D structure of the interaction between tsRNA and predicted genes, demonstrating the specific location of the binding site on the 3′UTR (mRNA) or the full length of a particular sequence. **(B)** GO enrichment analysis for the 14 differentially expressed tsRNAs. **(C)** KEGG pathway analysis for the 14 differentially expressed tsRNAs. GO, Gene ontology; KEGG, Kyoto Encyclopedia of Genes and Genomes database.

Increasing amounts of evidence has revealed that tsRNAs contain some seed sequences (positions 2–7 nt at the 5′ends) that could perform an miRNA-like mode of action and identify their mRNA targets by antisense pairing, inhibiting the expression level of the targets (Kumar et al., 2014; Kumar et al., 2016; Riffo-Campos et al., 2016) (Figure 4A). Among 16 differentially expressed tsRNAs verified above, we excluded 2 tsRNAs with the same seed sequence as the other sequences. Finally, 14 tsRNAs were selected for bioinformatics analysis (Table 2). And then, we used two algorithms (TargetScan and miRanda) to predict the mRNA targets of the 14 candidate tsRNAs. Combined with the two algorithms together, we obtained a total of 2,527 mRNA targets.

**TABLE 2:** The candidate tsRNAs selected for bioinformatics

tsRNA_ID	Type	tRFdb_ID	Length	logFC	padj	Regulation
tiRNA-1:33-Pro-CGG-1	tiRNA-5	-	33	2.667854	0.009664	Up
tRF-1:22-Ser-GCT-1-M4	tRF-5b	-	22	2.336799	0.05431	Up
tRF-58:75-Gln-CTG-1-M7	tRF-3a	3007a	18	3.320385	0.044557	Up
tRF-69:86-Leu-CAA-1-M5	tRF-3a	3018a	18	3.881438	0.01871	Up
tRF-1:16-Leu-TAG-3	tRF-5a	-	16	1.627943	0.041306	Up
tRF-1:22-Ser-AGA-1-M3	tRF-5b	-	22	3.491182	0.00942	Up
tRF-1:32-Gly-ACC-1	tRF-5c	-	32	1.950371	0.044087	Up
tRF-1:22-Lys-TTT-1	tRF-5b	-	22	1.949221	0.046665	Up
tRF-60:76-Tyr-GTA-1-M5	tRF-3a	3046a	17	3.22135	0.069064	Up
tRF-1:28-Gly-GCC-1	tRF-5c	-	28	−1.47745	0.017336	Down
tRF-1:23-Glu-CTC-1-M3	tRF-5b	5022b	23	−3.07916	0.001160	Down
tRF-1:28-Glu-TTC-2	tRF-5c	-	28	−1.81963	0.009118	Down
tRF-1:31-His-GTG-1	tRF-5c	-	31	−1.67124	0.015401	Down
tRF-1:14-Gly-CCC-1	tRF-5a	-	14	−2.34437	0.008618	Down

For further acknowledgment of the 14 candidate tsRNAs, Gene Ontology (GO) and Kyoto Encyclopedia of Genes and Genomes (KEGG) functional enrichment analyses were conducted to unveil their potential regulation functions. GO enrichment analysis covers three parts: biological process (BP), cellular component (CC), and molecular function (MF). As for BP and MF, the main pathways enriched were related to ion transmembrane transport and lipid metabolism. In terms of CC, these target genes were mainly response for cell membrane system, especially all kinds of organelle membrane (Figure 4B).

According to the KEGG enrichment analysis, Calcium signaling pathway (mmu04020), FoxO signaling pathway (mmu04068), cAMP signaling pathway (mmu04024), Cytokine-cytokine receptor interaction (mmu04060) were involved, and the top ten significant pathways were displayed in Figure 4C.

Besides, target gene prediction and enrichment analysis were also carried out for tRF-3a that specifically appeared after Cd exposure using the above method. Surprisingly, As for CC and MF, the main pathways enriched were related to ion transmembrane transport and cell membrane system, especially all kinds of organelle membrane. In terms of BP, no pathway was enriched (Supplementary Figure S1A). As for KEGG enrichment analysis, Calcium signaling pathway (mmu04020), Neuroactive ligand-receptor interaction (mmu04080), and Wnt signaling pathway (mmu04310) were involved, and the top ten significant pathways were displayed in Supplementary Figure S1B.

Ion channels are pore-forming proteins expressed in cell membranes that allow only specific ions to pass through and help maintain optimal ion concentrations (Goyal et al., 2017). Thus, ion channels help regulate cellular activity by maintaining a balance of ion concentrations and are intimately involved in almost all aspects of physiology such as excitation, signaling, gene regulation, secretion, and absorption (Bagal et al., 2013; Rubaiy, 2017). Therefore, ion channels are prerequisite for maintaining the normal functions of cell. In view of the bioinformatic anslysis of the target genes and the relevant literature, it is plausible to hypothesize that potential target genes of differentially expressed tsRNA in sperm may influence the permeability of cell membrane system, especially the various organelles membrane, by influencing various ion channels. Mitochondrion is a membrane-bound organelle found in the cytoplasm of eukaryotic cells, the primary function of which is to generate large quantities of energy to maintain the basic functions of cells. Lysosome is also a membrane-bound organelle, which contains a wide varity of hydrolytic enzymes that have the capability to break down many types of biomolecules. So, in the next exploration, we will focus on these two typical membrane-enclosed organelles.

### Effects of Cadmium Exposure on Progeny Embryo

After fertilization, Differentially expressed tsRNAs in sperm could be transmitted to zygotes and affect early embryonic development by regulating the expression of potential target genes. In view of the enriched pathways of the target genes, it is plausible to speculate that these 14 tsRNAs might affect cell membrane system, especially all kinds of organelle membrane, by regulating the ion channel complexus.

**FIGURE 5:**
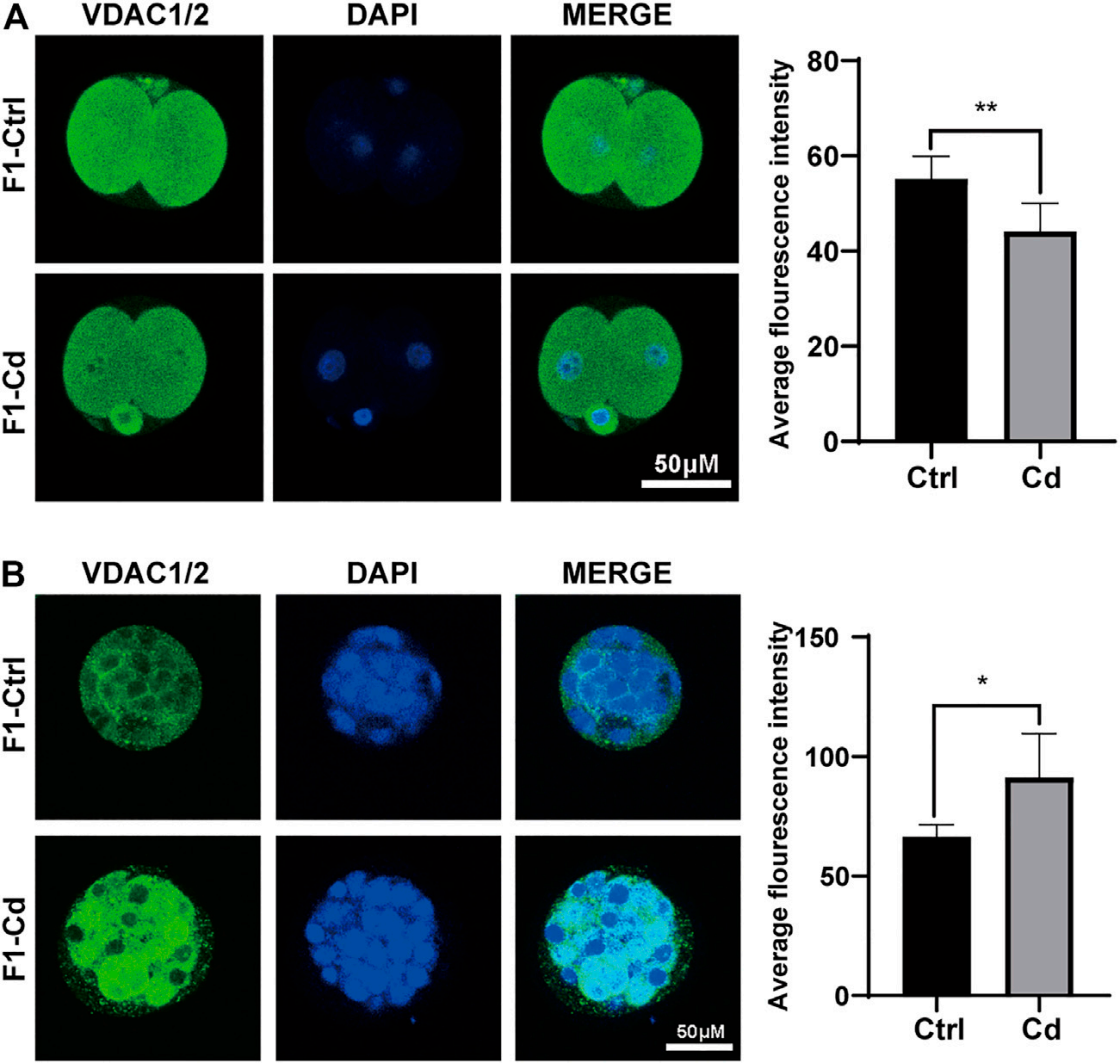
Effects of Cd exposure on progeny embryo. **(A)** The immunofluorescence and quantification analysis of VDAC1/2 in 2-cell embryos (scale bar = 50 μm, *n* = 5). **(B)** The immunofluorescence and quantification analysis of VDAC1/2 in morulas (scale bar = 50 μm, *n* = 5). All results are expressed as the mean ± SD. Labeled asterisk represents statistical significance compared with the respective control group. *p < 0.05, **p < 0.01.

Hence, we used embryo immunofluorescence to determine the amount of mitochondria in different stages of embryonic cells. We found that the mitochondrial content decreased in the two-cell stage (Figure 5A), but increased significantly in the morula stage (Figure 5B). As for the enhanced fluorescence of mitochondria in morula stage, it may be just a manifestation of enhanced mitochondrial activity to meet the needs of embryonic development. However, this question needs further experimental verification. However, whether the change of mitochondrial content in early embryonic cells continues into adulthood and has an effect on the progeny remains to be further observed.

### Effects of Cadmium Exposure on Progeny Testis

The role of cell membrane system in the structure and function of the male reproductive system has been an interesting and important research area. To investigate whether parental Cd exposure affects the structure and function of the cell membrane system in progeny testis, we used transmission electron microscopy, immunofluorescence and western blot to study the contents of mitochondria and lysosomes in testes at different periods.

**FIGURE 6:**
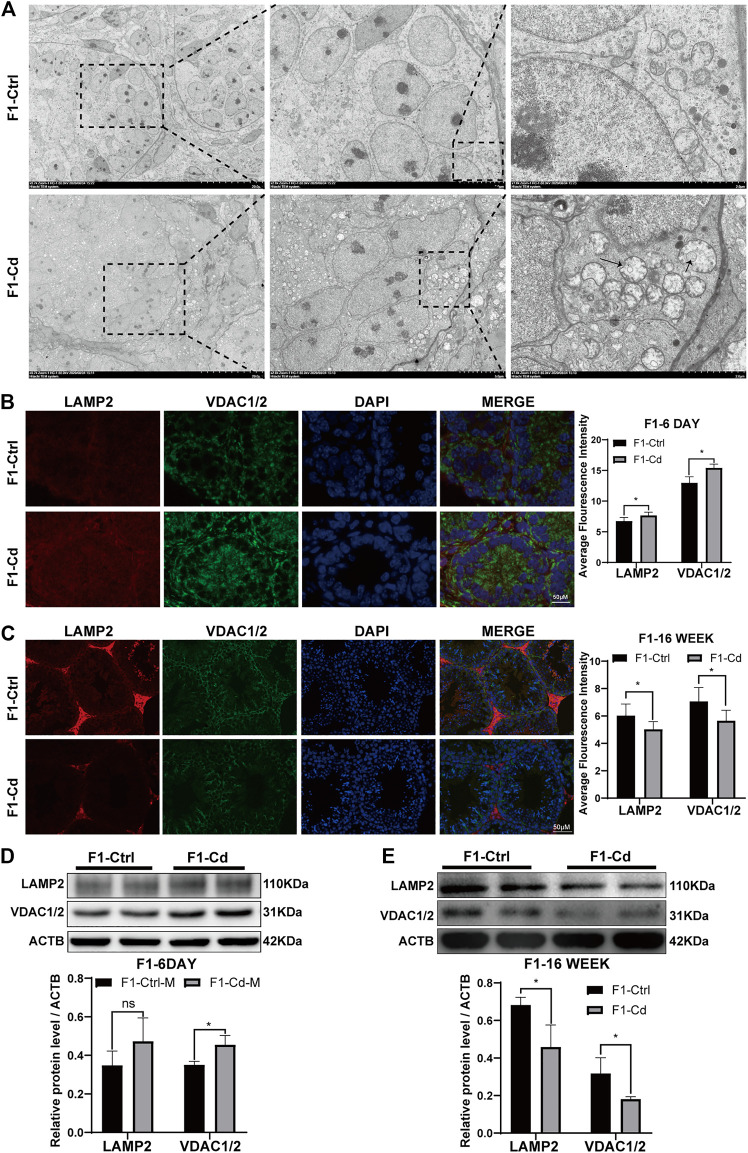
Effects of Cd exposure on progeny testis. **(A)** Transmission electron microscopy (TEM) results of the testes in mice. The black arrows manifest the mitochondrial vacuole, accompanied by the decreased mitochondrial cristae. **(B)** The immunofluorescence and quantification analysis of LAMP2 and VDAC1/2 in the testes of F1 mice at 6 days after birth (scale bar = 50 μm, *n* = 3). **(C)** The immunofluorescence and quantification analysis of LAMP2 and VDAC1/2 in the testes of F1 mice at 16 weeks (scale bar = 50 μm, *n* = 3). **(D)** The protein levels of LAMP2 and VDAC1/2 in mice testicular tissue at 6 days were measured by western blotting (*n* = 3). **(E)** The protein levels of LAMP2 and VDAC1/2 in mice testicular tissue at 16 weeks were measured by western blotting (*n* = 3). All results are expressed as the mean ± SD. Labeled asterisk represents statistical significance compared with the respective control group. *p < 0.05, **p < 0.01, ***p < 0.001 and ****p < 0.0001, ns, no significant difference.

First, the testes of 6 day-old mice were examined by transmission electron microscopy. Interestingly, it was found that the number of mitochondrion increased significantly in the type A spermatogonia, Sertoli cells and interstitial cells of F1-Cd group, accompanied by mitochondrial vacuolation (Figure 6A). Then, we used immunofluorescence to detect mitochondrial and lysosomal related molecular markers (VDAC1/2, LAMP2) to investigate the content of mitochondria and lysosomes. The results showed that the expression levels of VDAC1/2 and LAMP2 were significantly increased in the testes of F1-Cd mice, compared with that of the control group (Figure 6B). To verify this phenomenon, western blotting was further used to detect mitochondrial and lysosomal contents. In line with immunofluorescence results, western blotting result of VDAC1/2 showed increased mitochondrial contents in the F1-Cd group (Figure 6D). Although an increasing trend was observed in the western blotting result of LAMP2, there was no significant difference between the two groups (Figure 6D).

Next, the testes of 16 week-old mice were examined by immunofluorescence. Surprisingly, mitochondrial and lysosomal related markers (VDAC1/2 and LAMP2) in the F1-Cd group were significantly lower than those in the F1-Ctrl group (Figure 6C). To verify this phenomenon, western blotting was further used to detect mitochondrial and lysosomal contents. In line with immunofluorescence results, western blotting results of VDAC1/2 and LAMP2 also showed decreased mitochondrial and lysosomal contents in the F1-Cd group (Figure 6E).

Above all, these immunofluorescence and western blotting results in the testes of 16 week-old mice were inconsistent with those of 6 day-old mice. The possible explanation is that energy metabolism at different physiological stages is inconsistent, which leads to dynamic changes of mitochondrial and lysosomal contents. And it is strange that LAMP2 appeared to be concentrated in the testicular interstitial tissue of adult mice in both the F1-Cd and F1-Ctrl groups, while its signal appeared to be more ubiquitous in 6 day-old mice. This might be due to the increased demand for testosterone synthesis in adult male mice, resulting in increased levels of autophagy and lysosomes in the interstitial tissue. Lysosomes regulate testosterone synthesis involving various processes of autophagy (Gao et al., 2018; Alu et al., 2020; Zeng et al., 2021).

Collectively, these data indicated that paternal Cd exposure may influence progeny testis metabolism through cell membrane system.

### Effects of Cadmium Exposure on Progeny Liver

**FIGURE 7:**
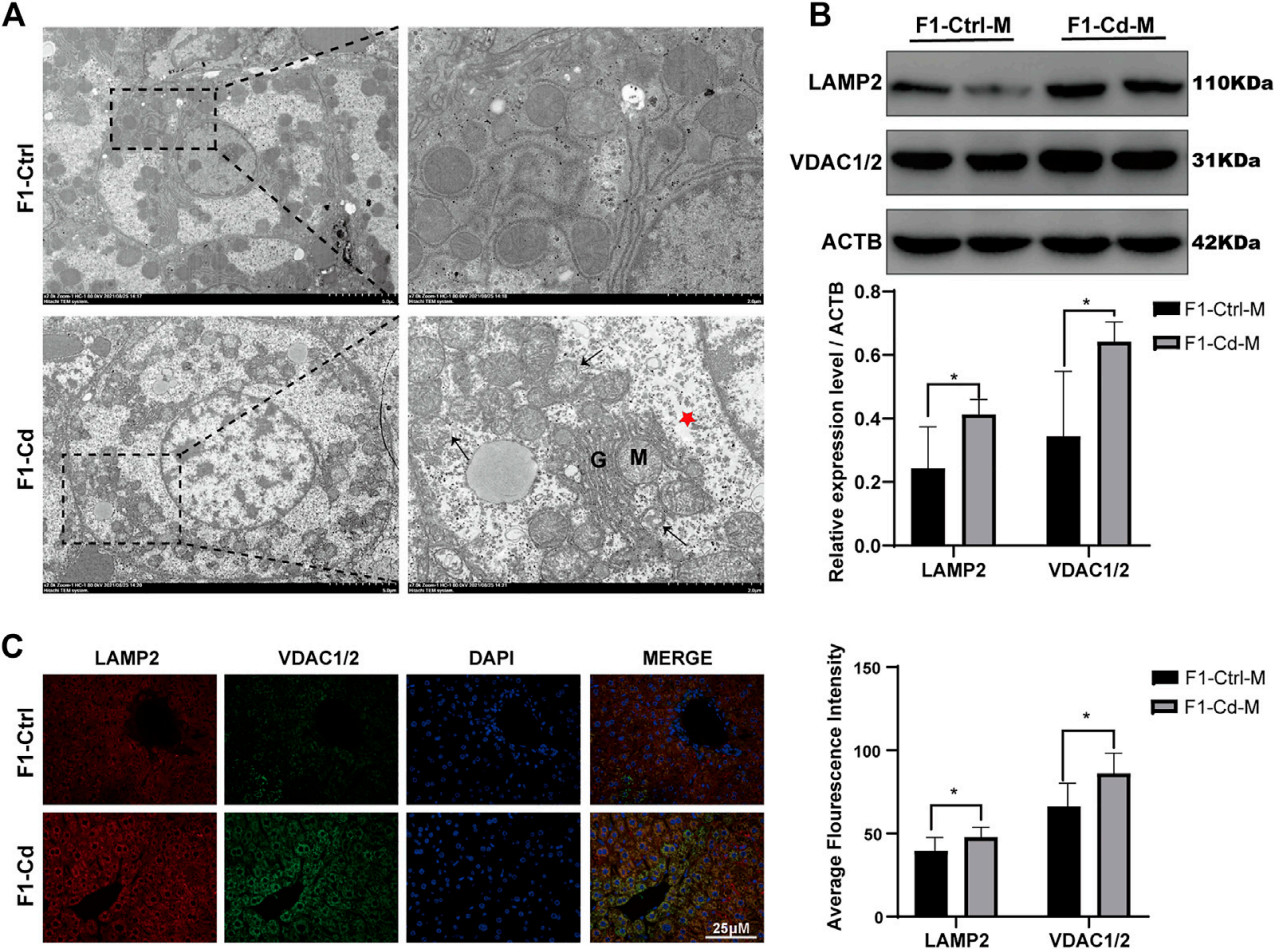
Effects of Cd exposure on progeny liver. **(A)** Transmission electron microscopy (TEM) results of the liver in mice. The red pentacle represents edema of the liver cells; the black arrows manifest the mitochondrial membrane rupture; M mitochondria; G, Golgi apparatus. **(B)** The protein levels of LAMP2 and VDAC1/2 in mice livers at 16 weeks were measured by western blotting (*n* = 3). **(C)** The immunofluorescence and quantification analysis of LAMP2 and VDAC1/2 in the livers of F1 mice at 16 weeks (scale bar = 25 μm, *n* = 3). All results are expressed as the mean ± SD. Labeled asterisk represents statistical significance compared with the respective control group. *p < 0.05, **p < 0.01, ***p < 0.001 and ****p < 0.0001.

Liver is an essential metabolic organ, which maintains energy metabolism homeostasis and are abundant in organelles (Rui, 2014). Therefore, we first used transmission electron microscopy to observed the organelles in the liver cells of 16 week-old male offspring of Cd-exposed group. Interestingly, we found that in the F1-Cd group, the hepatic cells were edema and the number of mitochondria increased accompanied by mitochondrial membrane rupture (Figure 7A). Then we used western blotting to detect the content of mitochondria and lysosomes. The results showed that the expression levels of VDAC1/2 and LAMP2 were significantly increased in the F1-Cd group (Figure 7B). This was consistent with the immunofluorescence results of VDAC1/2 and LAMP2 in the liver of F1-Cd group (Figure 7C). These data indicated that paternal Cd exposure may influence progeny liver metabolism through cell membrane system.

## Discussion

This study systematically revealed the changes of sperm tsRNA profiles after Cd exposure, which may provide new insights into the pathophysiology of Cd pollution. We found that there were 14 markedly changed tsRNAs (9 up-regulated and 5 down-regulated) in the mice sperm after Cd exposure, compared to the control group. Bioinformatics of tsRNA-mRNA-pathway interactions revealed the altered biological functions mainly were related to ion transmembrane transport, lipid metabolism and cell membrane system. Therefore, we focused on two stages of early embryo development and selected two organs to study the impact of these changes on offspring health. Surprisingly, we found that the contents of mitochondrion and lysosome were dynamically changed at different physiological stages and in different organs.

Cadmium (Cd) is a toxic heavy metal and ubiquitous environmental endocrine disruptor (EDC). Common EDCs, such as bisphenol A (BPA) (Chen et al., 2015; Mao et al., 2015; Dobrzyńska et al., 2018), Di-2-ethylhexyl phthalate (DEHP) (Dobrzyńska et al., 2012), 2,3,7,8-tetrachlorodibenzop-dioxin (TCDD) (Manikkam et al., 2012; Baker et al., 2014; Bruner-Tran et al., 2014; Sanabria et al., 2016), and atrazine (ATZ) (Hao et al., 2016), not only have adverse effects on the reproductive system of male animals, but also can cause reproductive harm to the offspring, especially the decrease of sperm quality. However, the specific mechanism of how epigenetic changes induced by these EDCs mediate intergenerational or transgenerational inheritance needs further exploration. It has been suggested that epigenetic markers in germ cells (which include DNA methylation, histone modifications, and small non-coding RNAs) may be the carrier of the intergenerational or transgenerational inheritance. However, on one hand, global DNA methylation is reprogrammed twice post-fertilization, and histone modifications are largely removed after fertilization and only re-established after implantation (Wang et al., 2018). It is therefore still a major puzzle how these epigenetic markers bypass epigenetic reprogramming, avoid RNA degradation, and initiate a cascade of molecular events to eventually affect offspring phenotypes during early embryonic development. On the other hand, the research about sperm tsRNA is blooming again, demonstrating that sperm tsRNAs as a type of paternal epigenetic information carrier mediate intergenerational or transgenerational inheritance of acquired traits (Chen et al., 2016; Sharma et al., 2016; Yan and Zhai, 2016). In our study, we focused on sperm tsRNA and explored the effects of Cd exposure on sperm tsRNA expression profile. Through sequencing and bioinformatics analysis, we explored the possible mechanism of intergenerational inheritance from the view of sperm tsRNA-induced changes in cell membrane system. The mechanism of intergenerational or transgenerational inheritance is so complex, and we could not rule out the possibility that multiple epigenetic mechanisms have involved or interacted in the process. Although the hypothesis that sperm tsRNA-induced changes in the cell membrane system are involved in intergenerational inheritance remains a tentative possibility and requires further confirmation, it is also attractive. Testing this hypothesis requires further microinjection of the differential tsRNAs into the zygote to observe its effect on cell membrane systems and progeny health.

Based on our sequencing and bioinformatics, after Cd exposure, these altered sperm tsRNAs mainly target ion channels. By maintaining a balance of ion concentrations inside and outside the membranes (Goyal et al., 2017), ion channels are intimately involved in almost all aspects of physiology such as excitation, signaling, gene regulation, secretion, and absorption (Bagal et al., 2013; Rubaiy, 2017). So that, it is plausible to hypothesize that potential target genes of differentially expressed tsRNA in sperm may influence the permeability of cell membrane system and further affect the physiological function of the organism. In addition, according to our enrichment analysis in Figure 4B, we found that the enriched pathways were related to various organelle membrane, for example, mitochondria. So in our experiment, we just focused on two typical organelles, mitochondria and lysosome. However, the effects of cell membrane system damage on the organism are very extensive, and could affect the physiological functions of all systems in the body. And that different tissues and organs have different tolerance to the membrane system damage. So in our subsequent studies, further research for the plasma membrane or other organelles should be conducted according to the progeny phenotype.

Besides, in Figures 5A,B, we found that the mitochondrial content decreased in the two-cell stage, but increased significantly in the morula stage. However, previous study (Thundathil et al., 2005) has suggested that once ovulation, mtDNA replication is suspended and organelles are divided into a single blastomere through several rounds of cell divisions. Despite multiple cell divisions, the mtDNA copy number of each embryo does not change until the blastocyst stage (day 5–6 embryo) (Kim et al., 2019). As for the enhanced fluorescence of mitochondria in morula stage, it may be just a manifestation of enhanced mitochondrial activity to meet the needs of embryonic development, rather than the proliferation of mitochondria. Of course, it is also possible that the damaged embryos are stressed to initiate mitochondrial replication prematurely at the morula or early blastocyst stage in order to meet the needs of embryonic development and prepare for embryo implantation. However, this question needs further experimental verification. At the same time, whether the change of mitochondrial content in early embryonic cells continues into adulthood and has effects on the progeny remains to be further observed.

Furthermore, the contents of mitochondria and lysosome in adult testis were inconsistent with those in 6 day-old testis and adult liver. The possible explanation is that energy metabolism in different organs and at different physiological stages is inconsistent, which leads to dynamic changes of mitochondrial and lysosomal contents. However, the specific mechanism remains to be further explored.

Overall, our study revealed the altered expression profile of sperm tsRNAs after Cd exposure, which might be involved in the regulation of different biological functions during early embryo development, and ultimately affecting offspring health. This information will contribute to further research on the intergenerational inheritance mechanism of Cd pollution. Future studies should decipher the specific phenotypes of the progeny after Cd exposure and the possible mechanism.

## Data Availability

The data presented in the study are deposited in the GEO repository, accession number GSE190236.

## Abbreviations

BP, biological process; CC, cellular component; Cd, cadmium; Dnmt2, DNA methyltransferase; EVs, extracellular vesicles; EDCs, endocrine disrupting chemicals; FC, fold change; GO, Gene Ontology; HFD, high-fat diet; KEGG, Kyoto Encyclopedia of Genes and Genomes; LPD, low-protein diet; miRNA, microRNA; MF, molecular function; piRNA, Piwi-interacting RNA; Pre-tRNAs, precursor tRNAs; sncRNA, small non-coding RNA; tiRNAs, tRNA-derived small RNAs; tiRNAs, tRNA halves; tiRNA-5, 5′-tRNA halves; tiRNA-3, 3′-tRNA halves; tRFs, tRNA fragments.
