# Supplementary figures and images for: Differential Expression Profiles and Potential Intergenerational Functions of tRNA-Derived Small RNAs in Mice After Cadmium Exposure
Source: Front Cell Dev Biol. 2022 Jan 3;9:791784. doi: 10.3389/fcell.2021.791784 (PMC8762212; doi:10.3389/fcell.2021.791784)

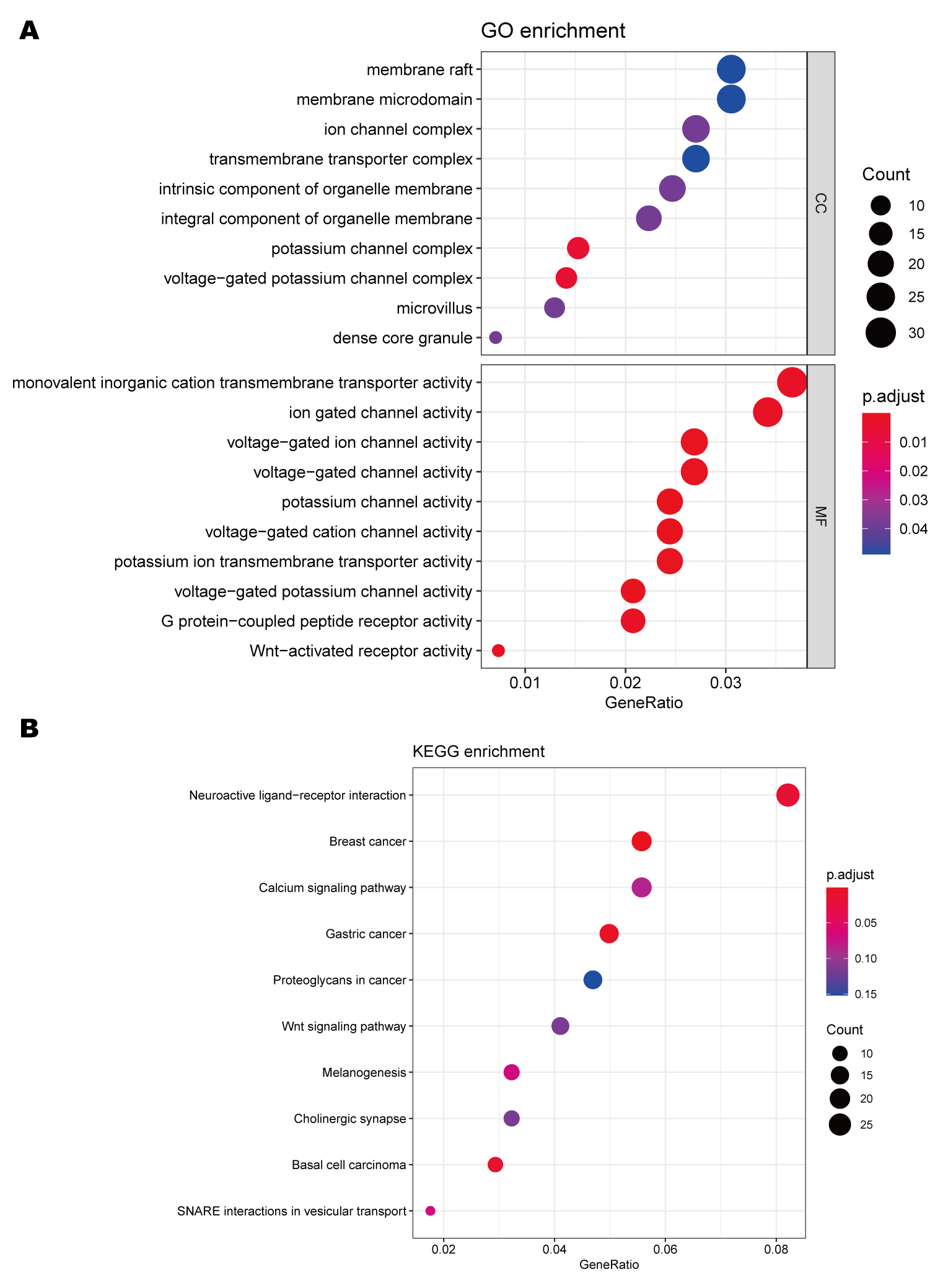

Supplement: Supplementary file 1 [file image1.tif]
