# Supplementary material for: Differential Expression Profiles and Potential Intergenerational Functions of tRNA-Derived Small RNAs in Mice After Cadmium Exposure
Source: Front Cell Dev Biol. 2022 Jan 3;9:791784. doi: 10.3389/fcell.2021.791784 (PMC8762212; doi:10.3389/fcell.2021.791784)
Supplement: Supplementary file 2 [file datasheet1.docx]

**Supplyment-Table 1 | The primer sequences of tsRNAs.**

| **Genes** | **Primer sequences (5′-3′)** | **AT (°C)** |
| --- | --- | --- |
| miRNA-specific forward primer | miRNA-specific forward primer provided by the The All-in-One™ miRNA qRT-PCR Detection Kit |  |
| tiRNA-1:33-Pro-CGG-1 | GGCTCGTTGGTCTAGGGGTATG | 58 |
| tRF-1:22-Ser-GCT-1-M4 | CGAGGTGGCCGAGTGGT | 58 |
| tRF-58:75-Gln-CTG-1-M7 | ATCTCGGTGGGACCTCCA | 58 |
| tRF-69:86-Leu-CAA-1-M5 | ATCCCACTTCTGACACCA | 56 |
| tRF-1:16-Leu-TAG-3 | GGTAGCGTGGCCGAGT | 56 |
| tRF-1:22-Ser-AGA-1-M3 | GTAGTCGTGGCCGAGTGGT | 60 |
| tRF-1:32-Gly-ACC-1 | GTTTCCGTAGTGTAGTGGTTAG | 54 |
| tRF-1:22-Lys-TTT-1 | GCCCGGATAGCTCAGTCGGTAG | 62 |
| tRF-60:76-Tyr-GTA-1-M5 | TCCGGCTCGAAGGACCA | 58 |
| tRF-1:31-Val-AAC-1-M2 | GCGAGTTTCCGTAGTGTAGTGGT | 60 |
| tRF-1:28-Gly-GCC-1 | GCATGGGTGGTTCAGTGGTAG | 58 |
| tRF-1:28-Glu-TTC-2 | TCCCATATGGTCTAGCGGTTAGGAT | 60 |
| tRF-1:31-His-GTG-1 | GCCGAGATCGTATAGTGGTTAG | 56 |
| tRF-1:14-Gly-CCC-1 | GCGAGCGCCGCTGGTGTA | 62 |
| tRF-1:29-Ala-CGC-5 | TCCCTGGTAGTCTAGTGGTTAGG | 58 |
| tiRNA-1:33-Gly-CCC-1 | GCGCCGCTGGTGTAGTG | 58 |
| tRF-1:30-Gly-TCC-1 | GCGTTGGTGGTATAGTGGTGAG | 58 |
| tRF-1:23-Glu-CTC-1-M3 | TCCCTGGTGGTCTAGTGGTTAG | 58 |
